# Impact of *KIR3DL1/3DS1* and *HLA-B* polymorphisms on hepatitis C virus infection: a case-control study

**DOI:** 10.3389/fcimb.2026.1801310

**Published:** 2026-05-12

**Authors:** Wen Yin, Tian Zeng, Chengrui Ren, Yuting Chen, Yue Guo, Kexin Ma, Yun Zhang, Rongbin Yu, Chongcai Wang, Jizheng Cui, Peng Huang, Ming Yue

**Affiliations:** 1 Department of Infectious Diseases, The First Affiliated Hospital with Nanjing Medical University, Nanjing, China; 2 Key Laboratory of Infectious Diseases, Department of Epidemiology and Biostatistics, School of Public Health, Nanjing Medical University, Nanjing, China; 3 Eastern Theater Command Centers for Disease Prevention and Control, Institute of Epidemiology and Microbiology, Nanjing, China; 4 Research and Development Department, Hainan International Travel Healthcare Center (Haikou Customs Port Clinic), Haikou, China; 5 Department of pain, Lianyungang Maternal and Child Health Hospital Affiliated to Kangda College of Nanjing Medical University, Lianyungang, China

**Keywords:** genetic polymorphism, hepatitis C virus, human leukocyte antigen, killer cell immunoglobulin & dashlike receptors, susceptibility

## Abstract

The polymorphism of killer-cell immunoglobulin-like receptors (KIRs) and human leukocyte antigen (HLA) genes determines the host’s susceptibility to viral infections. This study aimed to investigate the impact of five single nucleotide polymorphisms (SNPs) in *KIR3DL1/3DS1* and *HLA-B* genes on hepatitis C virus (HCV) infection. A total of 1881 individuals participated in this case-control study. SNPs including *KIR3DL1/3DS1* rs613491, rs605219, rs620977 and *HLA-B* rs3819288, rs1131170 were genotyped. Using multivariable logistic regression adjusting for established genetic and demographic confounders combined with stratified analysis and multidimensional bioinformatics analysis was employed to analyze the association between SNPs and HCV infection. Logistic regression analysis revealed that *KIR3DL1/3DS1* rs613491 CC and *HLA-B* rs1131170 CC were associated with increased susceptibility to HCV (codominant model: OR = 3.13, 95% CI = 1.90-5.17, *P* < 0.001; and OR = 4.20, 95% CI = 2.79-6.33, *P* < 0.001, respectively), whereas *HLA-B* rs1131170 AC showed a reduced susceptibility to HCV (codominant model: OR = 0.63, 95% CI = 0.49-0.81, *P* < 0.001). We speculate that the protective rs1131170-AC genotype may enhance either the quantity or quality of HCV-antigen presentation, thereby reducing susceptibility to infection. Stratified analysis indicated that rs613491 and rs1131170 also increased HCV susceptibility in certain subgroups. Bioinformatics analysis suggested the regulatory potential of these SNPs and their role in altering messenger RNA secondary structure, suggesting potential functional relevance that requires experimental validation in HCV susceptibility. Our findings demonstrate that *KIR3DL1/3DS1* rs613491 CC and *HLA-B* rs1131170 CC are significantly associated with increased HCV susceptibility, while rs1131170 AC is associated with reduced susceptibility.

## 1 Introduction

Hepatitis C virus (HCV) infection is a global health issue (Habib et al., 2023). According to World Health Organization (WHO) estimates, 50 million people live with chronic HCV infection. As a major causative agent of hepatocellular carcinoma (HCC), HCV promotes hepatocarcinogenesis through multiple mechanisms, including induction of epithelial–mesenchymal transition (EMT), dysregulation of cell cycle progression, and triggering of chronic inflammatory responses and tissue fibrosis, resulting in approximately 242000 deaths annually (Lin et al., 2025; Global hepatitis report 2024; action for access in low- and middle-income countries, 2024). With the advent of direct-acting antiviral drugs (DAAs), significant progress has been made in the treatment of HCV, achieving a sustained virological response (SVR) rate of over 95% (Zeng et al., 2023). However, the failure of DAA-cured individuals to acquire protective immunity, the risk of reinfection, and the bottlenecks in vaccine development all highlight the critical role of the host’s genetic background (Garbuglia et al., 2024). Therefore, conducting large-scale population studies to explore the association between host genetic factors and HCV infection may provide crucial scientific evidence for precision prevention strategies.

It is well known that the interaction between the host immune response and the virus is a decisive factor in the progression and outcome of infection (Goto et al., 2020). Natural killer (NK) cells also play a significant role in the immune response during chronic HCV infection (Gonzalez-Cao et al., 2018). They not only possess the ability to directly kill HCV-infected hepatocytes but also regulate adaptive immune responses, suppress viral replication, and activate other immune cells to participate in antiviral defense (Cho et al., 2018). Killer cell immunoglobulin-like receptors (KIRs) are a crucial class of receptors expressed on the surface of NK cells and belong to the immunoglobulin superfamily. KIRs bind to their corresponding ligands—human leukocyte antigen (HLA) class I molecules on the surface of target cells—to exert inhibitory or activating effects on NK cells (Parham and Guethlein, 2018). Under pathological conditions, such as in tumors, infections, or post-transplantation scenarios, abnormal or absent expression of HLA class I molecules on target cells leads to the loss of KIR ligands. This prevents NK cells from recognizing self-cells and weakens their inhibitory effects, resulting in NK cell activation and the killing of target cells (Dulberger et al., 2017). Therefore, the proteins encoded by *KIR/HLA* genes, as well as variations in these genes, play a vital regulatory role in the immune system.

The *KIR3DL1/3DS1* gene encodes highly polymorphic receptors expressed by NK cells and some T cells to modulate their effector functions in immunity (O’Connor and McVicar, 2013). These receptors interact with HLA class I ligands expressed on most nucleated cells to signal their health status to the immune system (Quatrini et al., 2021). The KIR3DL1 allotype is an inhibitory receptor that specifically binds to highly polymorphic HLA-A and -B subsets (Colonna and Samaridis, 1995). The KIR3DS1 allotype is an activating receptor that targets the non-polymorphic HLA-F and a smaller subset of HLA-A and -B molecules (Kiani et al., 2019). However, these findings remain inconsistent: Umemura T et al. found that in the Japanese population, carriers of the *KIR3DL1* and its ligand *HLA-Bw4* genotypes may be associated with the development of hepatocellular carcinoma (HCC) in HCV-induced cirrhosis (Umemura et al., 2021), while De Re V et al. observed that a reduced frequency of *HLA-Bw4*+*KIR3DS1*+ in the Italian population was linked to an increased risk of HCC (De Re et al., 2015). Lunemann S et al. demonstrated in cell culture systems, humanized liver mice, and primary liver tissues from HCV-infected individuals that *KIR3DS1* and its ligand *HLA-F* were upregulated in HCV-infected cells, and their interaction contributed to NK cell-mediated control of HCV (Lunemann et al., 2018). Thöns C et al. studied 266 German and 342 American people who inject drugs and discovered that the *KIR3DL1*/*HLA-Bw4–*80 genotype was associated with spontaneous HCV clearance (Thöns et al., 2017). However, Podhorzer A et al. reported no significant relationship between *KIR3DL1* and HCV infection outcomes (Podhorzer et al., 2017). These discrepancies likely reflect population-specific genetic backgrounds, varying routes of exposure, and differential viral genotype distributions.

In our previous studies, we found that single nucleotide polymorphisms (SNPs) of *KIR2DS4* rs35440472 and *HLA-C* rs1130838, *KIR2DL4* rs660773 and *HLA-G* rs9380142, as well as *KIR3DL2* rs11672983 and *KIR3DL2* rs3745902, were associated with increased susceptibility to HCV infection (Shen et al., 2021; Feng et al., 2023; Li et al., 2024). Therefore, based on these findings and unresolved questions, this study aims to explore the genetic association between five potentially functional SNPs in *KIR3DL1/3DS1/HLA-B* and HCV infection. The candidate SNPs include: *KIR3DL1/3DS1* rs613491, *KIR3DL1/KIR3DS1* rs605219, *KIR3DL1/3DS1* rs620977, *HLA-B* rs3819288, *HLA-B* rs1131170.

To minimize potential confounding effects from established genetic factors, we incorporated Interleukin 28B (IL28B, also known as IFNL4) into our analysis, given its well-documented association with HCV infection and treatment response (Medina et al., 2023). Notably, *IL28B* rs12979860 and *IL28B* rs8099917 have been significantly linked to spontaneous viral clearance and therapeutic outcomes in pegylated interferon-alpha/ribavirin (peg-IFN-α/RBV) regimens (da Silva et al., 2021). Consequently, these two single-nucleotide polymorphisms (SNPs) were genotyped and included as covariates in the final statistical models to account for the known influence of IL28B variants on HCV infection outcomes. We anticipate that this study will offer novel insights into host genetic determinants modulating immune responses to HCV infection.

It is critical to distinguish between susceptibility to infection (transition from uninfected to infected state, influenced by initial viral entry and innate immune recognition) and disease outcome (spontaneous clearance versus chronic persistence, influenced by adaptive immune responses and viral evolution). This study specifically focuses on the former, examining genetic variants associated with the risk of acquiring HCV infection among exposed individuals.

## 2 Materials and methods

### 2.1 Subjects

Between 2011 and 2018, this study enrolled a total of 1881 high-risk individuals for HCV infection, comprising 1440 paid blood donors (PBDs) from 20 villages in Jiangsu Province, and 441 dialysis patients (DPs) recruited from 9 hospitals. Inclusion criteria participants were eligible if they met the following conditions: no prior treatment with DAAs or interferon-based therapy before enrollment; minimum follow-up duration of 6 months; provision of written informed consent for voluntary participation. The exclusion criteria for the study subjects are as follows: unclear history of drug use or blood donation; co-infection with HIV or HBV; age <18 or >80 years; presence of autoimmune, alcoholic, or metabolic liver diseases. Participants were categorized into three groups: Group A (uninfected controls): HCV antibody negative and HCV RNA negative, with HCV RNA loads consistently below 30 IU/mL in two independent blood samples collected six months apart; Group B (spontaneous clearance group): HCV antibody positive and HCV RNA positive, with HCV RNA loads consistently above 30 IU/mL in two independent blood samples collected six months apart; Group C (persistent infection group): HCV antibody positive but HCV RNA negative, with HCV RNA loads consistently below 30 IU/mL in two independent blood samples collected six months apart. During the 6-month observation period, HCV status was confirmed using at least three different serological assays. Only samples with concordant results were included in the final analysis. A structured questionnaire, designed by professionals, was used to collect: Demographic details, High-risk behavior exposures, Laboratory test results, and Clinical history of HCV infection.

We combined Group B (spontaneous clearance) and Group C (persistent infection) into a unified “HCV-infected” cohort for susceptibility analysis based on the following rationale (1): Both groups share the critical transition from uninfected to infected state, which is the primary focus of this study—identifying genetic determinants of initial infection susceptibility rather than disease progression outcomes (2); Sample size considerations: Separate analysis of spontaneous clearance (n=311) would lack statistical power to detect moderate genetic effects (3); Biological plausibility: The KIR-HLA axis primarily regulates NK cell-mediated innate immune recognition of infected cells during the acute phase, which is relevant to both establishment and early control of infection. We acknowledge that this grouping strategy precludes analysis of genetic factors associated with spontaneous clearance versus chronic persistence, which we explicitly address as a limitation. This case-control design compares uninfected controls (Group A) with all individuals who became infected regardless of subsequent outcome (Groups B+C), thereby targeting the susceptibility phenotype rather than disease resolution phenotype.

### 2.2 Serological testing

Fasting venous blood (5–10 mL) was collected from participants in EDTA anticoagulant tubes. Plasma was separated by centrifugation and stored at -80 °C until analysis. HCV-specific antibodies were detected using a quantitative enzyme-linked immunosorbent assay (Diagnostic Kit for Antibody to HCV 3.0 ELISA; Intec Products Inc.). HCV RNA levels were quantified using real-time fluorescence quantitative PCR, with a detection limit of 20 IU/mL (HCV Nucleic Acid Quantification Test Kit; Daan Gene Co., Ltd.). HCV genotypes were detected using the Murex HCV Serotyping Assay ELISA Kit (Abbott) (Bhattacherjee et al., 1995).

### 2.3 Candidate SNP selection and genotyping

Candidate *KIR3DL1*/*3DS1*/*HLA-B* tagSNP were retrieved from the 1000 Genomes Project SNP data set (37.0 version) and selected using the Haploview software (version 4.2). The selection criteria of tagSNP included: a minor allele frequency ≥ 0.05 and a pairwise LD r^2^ ≥ 0.8 within the Chinese Han population. Considering the factors mentioned above, the following five SNPs were finally chosen as candidate SNPs for the further study: *KIR3DL1/3DS1* rs613491, *KIR3DL1/3DS1* rs605219, *KIR3DL1/3DS1* rs620977, *HLA-B* rs3819288, *HLA-B* rsrs1131170. The basic information of candidate SNPs can be found in **Supporting Information**: **Supplementary Table 1**.

Genotyping was performed using TaqMan allele discrimination assays on the Roche LightCycler^®^ 480 II Real-Time PCR System following standardized protocols. Primer and probe sequences for all SNPs are provided in **Supporting Information**: **Supplementary Table 2**. All genotyping procedures were conducted with the following quality control measures (1): Blinded sample processing to eliminate bias, achieving >90% genotyping success rate; (2) Random re-genotyping of 10% samples showing 100% concordance; (3) Strict adherence to manufacturer’s protocols with uniform procedures and instrumentation across all samples. Data analysis was performed using LightCycler^®^ 480 software (version1.5.1) with automated allele calling.

### 2.4 In silico analysis

To systematically evaluate the functional consequences of the single nucleotide polymorphisms (SNPs), we conducted comprehensive bioinformatics analyses using multiple established resources. SNP functional predictions were performed using the SNP Function Prediction tool (FuncPred; https://snpinfo.niehs.nih.gov/) and HaploReg version 4.2 database (https://pubs.broadinstitute.org/mammals/haploreg/haploreg.php). We assessed the potential regulatory functions of these SNPs through RegulomeDB scoring (RegulomeDB; http://www.regulomedb.org/). For RNA structural analysis, we employed the Vienna RNA Web Services’ RNAfold server (http://rna.tbi.univie.ac.at/) to predict SNP-induced alterations in messenger RNA (mRNA) secondary structures. The University of California Santa Cruz (UCSC) Genome Browser (http://genome.ucsc.edu/cgi-bin/hgGateway) was utilized to investigate the potential biological functions of these SNPs. Furthermore, we analyzed data from the Encyclopedia of DNA Elements (ENCODE) project to examine the expression patterns of two critical histone modifications: histone H3 lysine 4 monomethylation (H3K4me1) and histone H3 lysine 27 acetylation (H3K27ac). These analyses were performed across seven well-characterized cell lines: human embryonic stem cells (H1-hESC), human skeletal muscle myoblasts (HSMM), human umbilical vein endothelial cells (HUVEC), chronic myelogenous leukemia cells (K562), normal human epidermal keratinocytes (NHEK), lymphoblastoid cells (GM12878), and normal human lung fibroblasts (NHLF). This multi-dimensional approach enabled us to elucidate the role of SNPs in gene regulation and cell identity maintenance, while also revealing their context-specific functional variations across different cellular and biological systems.

### 2.5 Statistical analysis

Hardy-Weinberg equilibrium (HWE) was tested separately within uninfected controls stratified by population subgroup: dialysis patients (DP, n=325) and paid blood donors (PBD, n=700), as well as in the combined control group (n=1025). All five candidate SNPs were consistent with HWE in the overall control population, as well as rs613491 and rs1131170 in both subgroups (all *P*>0.05; **Supporting Information**: **Supplementary Tables 2**, **3**).

Comparative analyses of demographic characteristics, clinical parameters, and virological data were performed using appropriate statistical tests: χ² test for categorical variables, one-way ANOVA for normally distributed continuous variables, and Kruskal-Wallis test for non-parametric data. We conducted multivariable logistic regression analyses adjusted for potential confounders including gender, age, route of infection, as well as genotypes of *IL28B* rs12979860 and *IL28B* rs8099917. These analyses evaluated associations between candidate SNPs and HCV infection status with odds ratio (OR) and 95% confidence interval (CI). HCV genotype was only considered as a covariate in the spontaneous clearance analysis (Group B vs. Group C), where it is biologically relevant to disease outcome. For comprehensive genetic assessment, we implemented four inheritance models: codominant, dominant, additive and recessive frameworks. Bonferroni correction was used to correct for multiple comparisons, and the *P* Value was adjusted to 0.01 (0.05/5). All statistical computations were performed using IBM SPSS Statistics (Version 26.0).

## 3 Results

### 3.1 Demographic and clinical characteristics

The demographic and clinical characteristics of the participants are detailed in **Table 1**. The study encompassed a total of 1881 subjects, with 1025 individuals in Group A, 311 in Group B, and 545 in Group C. There were statistically significant differences among the three groups regarding gender, age, ALT, AST, infection routes, and the distribution of HCV genotypes (all *P* < 0.001). However, no significant differences were observed in the gene phenotype distributions of *IL28B* rs12979860 and *IL28B* rs8099917 across the three cohorts (all *P* > 0.05). In the control group, all five candidate SNPs adhered to the Hardy-Weinberg equilibrium (all *P* > 0.05).

**Table 1:** Demographic and clinical characteristics among HCV control, spontaneous clearance and persistent infection groups.

Variables	Group A n (%) n=1025	Group B n (%) n=311	Group C n (%) n=545	*P*
Age (mean ± SD)	58.14 ± 11.48	57.58 ± 8.29	55.58 ± 8.97	
<50	236 (23.0)	49 (15.8)	154 (28.3)	**<0.001** ^a^
≥50	789 (77.0)	262 (84.2)	391 (71.7)	
Gender				
Male	389 (38.0)	81 (26.0)	148 (27.2)	**<0.001** ^a^
Female	636 (62.0)	230 (74.0)	397 (72.8)	
ALT (U/L)				
<50	999 (97.9)	263 (84.8)	366 (67.4)	**<0.001** ^a^
≥50	21 (2.1)	47 (15.2)	177 (32.6)	
AST (U/L)				
<40	981 (96.3)	243 (78.4)	317 (58.4)	**<0.001** ^a^
≥40	38 (3.7)	67 (21.6)	226 (41.6)	
Routes of infection				
DP	325 (31.7)	65 (20.9)	51 (9.4)	**<0.001** ^a^
PBD	700 (68.3)	246 (79.1)	494 (90.6)	
HCV genotype				
1	–	32 (45.1)	128 (39.1)	**<0.001** ^b^
Non-1	–	21 (29.6)	12 (3.7)	
Mixed	–	18 (25.4)	187 (57.2)	
*IL28B*-rs12979860				
CC	919 (89.9)	282 (91.6)	482 (88.8)	0.429^a^
CT/TT	103 (10.1)	26 (8.4)	61 (11.2)	
*IL28B*-rs8099917				
TT	888 (88.2)	280 (90.3)	483 (89.0)	0.573^a^
TG/GG	119 (11.8)	30 (9.7)	60 (11.0)	

Non‐1: HCV genotypes other than genotype 1, such as genotype 2, genotype 3, genotype 4, genotype 5, and genotype 6. Mixed: Coexistence of two or more HCV genotypes at the same time. Bold type indicates statistically significant results.ALT, alanine aminotransferase; AST, aspartate aminotransferase; Group A, uninfected control group; Group B, spontaneous clearance group; Group C, persistent infection group; HCV, hepatitis C virus; IL28B, Interleukin 28B; DP, dialysis population; PBD, paid blood donors.

^a^*P* Value of χ^2^‐test among two groups; ^b^*P* Value of χ^2^‐test among three groups.

Bolded text represents substantially significant outcomes.

### 3.2 Association of candidate SNPs with the susceptibility to HCV infection

To investigate the association between single nucleotide polymorphisms (SNPs) in the candidate genes *KIR3DL1/3DS1/HLA-B* and susceptibility to hepatitis C virus (HCV), we combined subjects from groups B and C into a unified HCV-infected cohort and compared them with the uninfected control group A. The genotype distribution of the five SNPs is detailed in **Table 2**. After adjusting for confounding variables such as age, sex, *IL28B* rs12979860, *IL28B* rs8099917, and route of infection, logistic regression analysis showed that compared with wild-type TT genotype carriers, individuals carrying the rs613491 CC genotype had increased susceptibility to HCV infection (adjusted OR = 3.13, 95%CI=1.90-5.17, *P* < 0.001); compared with wild-type AA genotype carriers, individuals carrying the rs1131170 AC genotype had decreased susceptibility to HCV infection (adjusted OR = 0.63, 95%CI=0.49-0.81, *P* < 0.001), while individuals carrying the rs1131170 CC genotype had increased susceptibility to HCV (adjusted OR = 4.20, 95%CI=2.79-6.33, *P*<0.001). Additionally, regression genetic model analysis also yielded similar results: *KIR3DL1/3DS1* rs613491: Recessive model: OR = 3.09, 95% CI = 1.88-5.08, *P* < 0.001; Additive model: OR = 1.35, 95% CI = 1.13-1.60, *P* < 0.001; *HLA-B* rs1131170: Recessive model: OR = 4.68, 95% CI = 3.12-7.04, *P* < 0.001; Additive model: OR = 1.34, 95% CI = 1.14-1.57, *P* < 0.001. It is worth noting that after using Bonferroni correction for multiple comparisons, they remained significant.

**Table 2:** Genotypic distribution of *KIR3DL1/3DS1* and *HLA-B* genes among control, spontaneous clearance and persistent infection groups.

Gene	SNPs (genotype)	Group A n (%) n=1025	Group B n (%) n=311	Group C n (%) n=545	Group (B+C) n (%) n=856	OR (95%CI)^a^	*P* ^a^	OR (95%CI)^b^	*P* ^b^
*KIR3DL1/3DS1*	rs613491								
	TT	755 (74.1)	213 (71.7)	357 (69.2)	570 (70.1)	1		1	
	TC	237 (23.3)	66 (22.2)	122 (23.6)	188 (23.1)	1.06 (0.84-1.33)	0.640	1.10 (0.77-1.57)	0.589
	CC	27 (2.6)	18 (6.1)	37 (7.2)	55 (6.8)	**3.13 (1.90-5.17)**	**<0.001**	1.59 (0.86-2.95)	0.143
	Dominant model					1.25 (1.01-1.55)	0.042	1.20 (0.86-1.66)	0.282
	Recessive model					**3.09 (1.88-5.08)**	**<0.001**	1.55 (0.84-2.86)	0.161
	Additive model					**1.35 (1.13-1.60)**	**<0.001**	1.19 (0.93-1.53)	0.163
*KIR3DL1/3DS1*	rs605219								
	TT	341 (33.9)	87 (30.7)	167 (34.6)	254 (33.2)	1		1	
	TC	513 (50.9)	168 (59.4)	276 (57.1)	444 (58.0)	0.99 (0.80-1.24)	0.949	0.78 (0.56-1.10)	0.158
	CC	153 (15.2)	28 (9.9)	40 (8.3)	68 (8.9)	0.70 (0.49-0.99)	0.044	0.71 (0.40-1.28)	0.252
	Dominant model					0.93 (0.75-1.15)	0.503	0.77 (0.56-1.08)	0.126
	Recessive model					0.69 (0.50-0.97)	0.033	0.82 (0.48-1.42)	0.488
	Additive model					0.88 (0.75-1.03)	0.121	0.82 (0.63-1.06)	0.128
*KIR3DL1/3DS1*	rs620977								
	AA	751 (73.7)	222 (72.1)	371 (69.9)	593 (70.7)	1		1	
	AT	254 (24.9)	84 (27.3)	159 (29.9)	243 (29.0)	1.24 (1.00-1.54)	0.049	1.21 (0.87-1.67)	0.254
	TT	14 (1.4)	2 (0.6)	1 (0.2)	3 (0.4)	0.24 (0.07-0.85)	0.027	0.26 (0.02-2.90)	0.273
	Dominant model					1.18 (0.96-1.47)	0.120	1.18 (0.86-1.63)	0.309
	Recessive model					0.23 (0.06-0.80)	0.021	0.25 (0.02-2.75)	0.255
	Additive model					1.10 (0.90-1.35)	0.340	1.14 (0.84-1.57)	0.402
*HLA-B*	rs3819288								
	TT	545 (54.4)	156 (51.5)	290 (55.6)	446 (54.1)	1		1	
	TC	382 (38.1)	122 (40.3)	193 (37.0)	315 (38.2)	0.99 (0.81-1.21)	0.908	0.83 (0.61-1.13)	0.241
	CC	75 (7.5)	25 (8.3)	39 (7.5)	64 (7.8)	1.06 (0.73-1.54)	0.765	0.82 (0.47-1.44)	0.494
	Dominant model					1.00 (0.82-1.21)	0.995	0.83 (0.62-1.11)	0.213
	Recessive model					1.06 (0.74-1.53)	0.738	0.89 (0.52-1.53)	0.671
	Additive model					1.01 (0.87-1.18)	0.893	0.87 (0.69-1.10)	0.248
*HLA-B*	rs1131170								
	AA	712 (69.5)	193 (66.1)	368 (73.2)	561 (70.6)	1		1	
	AC	274 (26.7)	47 (16.1)	81 (16.1)	128 (16.1)	**0.63 (0.49-0.81)**	**<0.001**	0.92 (0.61-1.40)	0.704
	CC	39 (3.8)	52 (17.8)	54 (10.7)	106 (13.3)	**4.20 (2.79-6.33)**	**<0.001**	0.62 (0.40-0.97)	0.034
	Dominant model					1.04 (0.84-1.29)	0.723	0.77 (0.56-1.07)	0.120
	Recessive model					**4.68 (3.12-7.04)**	**<0.001**	0.63 (0.41-0.97)	0.037
	Additive model					**1.34 (1.14-1.57)**	**<0.001**	0.81 (0.66-1.00)	0.047

*P* Value, OR, and 95% CIs of ^<s> (</s>a)^Group (B + C) Ver A where ^(b)^for Group C Ver B, were computed on the basis of the logistic regression model, adjusted by sex, age, *IL28B* rs12979860, *IL28B* rs8099917, and infection route. Bolded text represents substantially significant outcomes.Group A, uninfected control group; Group B, spontaneous clearance group; Group C, persistent infection group; HLA, human leukocyte antigen; IL28B, Interleukin 28B; KIR, killer‐cell immunoglobulin‐like receptors; SNPs, single nucleotide polymorphisms.

Bolded text represents substantially significant outcomes.

The two significant SNPs (rs613491 and rs1131170) were further examined in stratified analyses to control for confounding variables, including sex, age and route of infection, which may introduce bias. Compared with carriers of the wild-type alleles of *KIR3DL1/3DS1* rs613491 and *HLA-B* rs1131170, carriers of the mutant alleles of these SNPs had significantly increased risk in some subgroups (all *P* < 0.05, showed in **Table 3**).

**Table 3:** Stratified analysis of the association of rs613491, rs1131170 with HCV susceptibility.

Gene	SNPs	Subgroups	Group A	Group (B+C)	OR (95%CI)*^a^*	*P^a^*
			n (WW+WMvsMM)	n (WW+WMvsMM)		
*KIR3DL1/3DS1*	rs613491	Age				
		<50	233/2	176/10	**9.54 (1.85-49.05)**	**0.007**
		≥50	759/25	582/45	**2.57 (1.53-4.30)**	**<0.001**
		Gender				
		Male	377/10	204/16	**3.37 (1.47-7.72)**	**0.004**
		Female	615/17	554/39	**2.63 (1.41-4.88)**	**0.002**
		Routes of infection				
		DP	313/8	94/15	**5.51 (2.23-13.58)**	**<0.001**
		PBD	679/19	664/40	2.02 (1.14-3.57)	0.016
*HLA-B*	rs1131170	Age				
		<50	225/11	168/19	3.19 (1.32-7.69)	0.010
		≥50	761/28	521/87	**4.93 (3.12-7.79)**	**<0.001**
		Gender				
		Male	374/15	175/38	**5.57 (2.94-10.58)**	**<0.001**
		Female	612/24	514/68	**4.20 (2.47-7.15)**	**<0.001**
		Routes of infection				
		DP	309/16	80/24	**5.78 (2.86-11.66)**	**<0.001**
		PBD	677/23	609/82	**4.16 (2.54-6.81)**	**<0.001**

Bold type indicates statistically significant results.HCV, hepatitis C virus; OR, odds ratio; CI, confidence interval; Group A, uninfected control group; Group B, spontaneous clearance group; Group C, persistent infection group; Group (B + C): infected group; DP, dialysis population; PBD, paid blood donors; KIR, killer‐cell immunoglobulin‐like receptors; HLA, human leukocyte antigen; M, mutant‐type allele; W, wildtype allele.

^a^ *P* Value, OR and 95% CIs of Group A versus Group (B + C) were calculated based on the logistic regression model, adjusted by gender, age, *IL28B* rs12979860, *IL28B* rs8099917 and routes of infection;.

Bolded text represents substantially significant outcomes.

### 3.3 Correlation between candidate SNPs and the spontaneous clearance of HCV infection

We analyzed the association between candidate SNPs and HCV spontaneous clearance by comparing SNP distribution frequencies between Group B (spontaneous clearance group) and Group C (persistent infection group). However, logistic regression analysis using four genetic models (adjusted for sex, age, *IL28B* rs12979860, *IL28B* rs8099917 and infection routes) revealed no significant associations between any of the five candidate SNPs (*KIR3DL1/3DS1* rs613491, *KIR3DL1/3DS1* rs605219, *KIR3DL1/3DS1* rs620977, *HLA-B* rs3819288, *HLA-B* rs1131170) and HCV spontaneous clearance (all *P* > 0.05, **Table 2**). Consequently, no further stratified analysis was performed on these data. Due to missing HCV genotype data in some cases, it was not feasible to include this variable in the logistic regression analysis. Sensitivity analyses adjusting for HCV genotype yielded consistent null findings (all *P* > 0.05), confirming the absence of association between these SNPs and spontaneous clearance (**Supporting Information**: **Supplementary Table 6**).

### 3.4 Bioinformatics analysis

**Figure 1:**
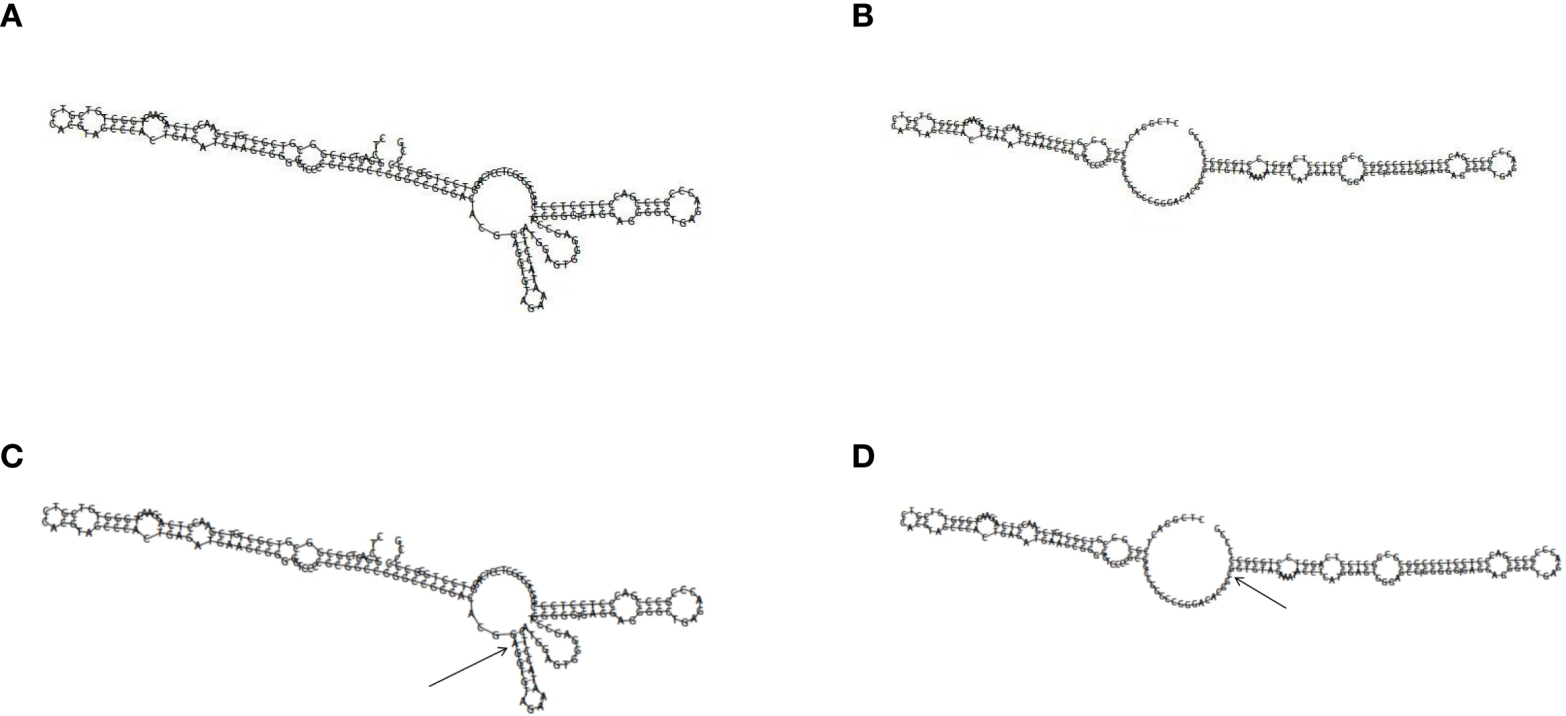
The impact of rs1131170 on HLA-B mRNA optimal secondary structures. Minimum free energy of the mRNA optimal secondary structure for **(A, C)** rs1131170-A (MFE = −92.60 kcal/mol) and **(B, D)** rs1131170-C (MFE = −90.40 kcal/mol). The RNAfold web server demonstrated modifications to the local structure. The arrows in **(C)** and **(D)** indicate the mutation's location. HLA, human leukocyte antigen; mRNA, messenger RNA. Abbreviations: HLA, human leukocyte antigen; MFE, minimum free energy; mRNA, messenger RNA.

**Figure 2:**
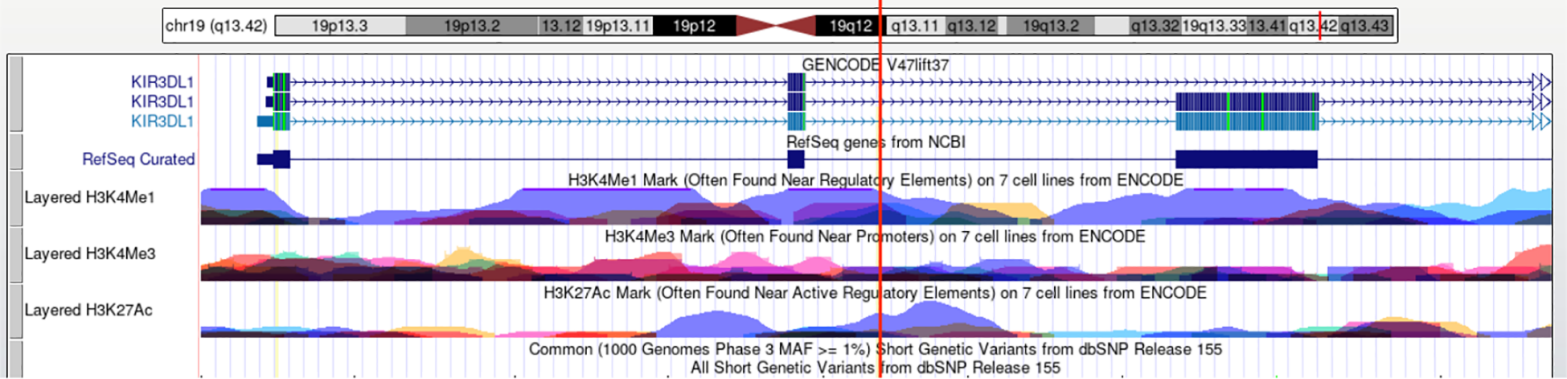
Functional annotation for *KIR3DL1/3DS1* rs613491 using ENCODE data from UCSC genome browser. The histone H3 lysine 4 monomethylation (H3K4Me1) and lysine 27 acetylation (H3K27Ac) marker in 7 cell lines (GM12878, H1‐hESC, HSMM, HUVEC, K562, NHEK, and NHLF cells) as well as HepG2 liver cell line are presented. The red line indicates the position of SNP rs613491 (Data available: http://genome.ucsc.edu/). ENCODE, Encyclopedia of DNA Elements; UCSC, University of California Santa Cruz.

In silico prediction suggests that *HLA-B* rs1131170 may function as exonic splicing enhancers or silencers (ESE/ESS) and nonsynonymous SNPs (nsSNPs). RegulomeDB scores for rs1131170 are “1f, “ indicating potential involvement in expression quantitative trait loci (eQTL) or chromatin accessibility quantitative trait loci (caQTL). These loci could influence gene expression or chromatin accessibility in specific regions, such as chromatin open peaks. Furthermore, the impact of mutations at the rs1131170 loci on the secondary structure of *HLA-B* mRNA was analyzed using the RNAfold web server. The results, depicted in **Figure 1**, reveal significant differences between the wild type and mutant type of rs1131170, indicating alterations in the *HLA-B* mRNA secondary structure. In addition, the potential biological functions of rs613491 were annotated in the ENCODE project using the UCSC genome browser. Rs613491 was found to be located on elevated peaks of H3K4Me3 and H3K27Ac markers in seven cell lines. This positioning was confirmed by the enrichment of H3K4Me3 and H3K27Ac observed in CHIP-seq assays (**Figure 2**). These bioinformatics analyses are predictive and do not provide direct experimental evidence; functional validation through luciferase reporter assays, EMSA, or NK cell functional assays is required to confirm these hypotheses.

### 3.5 Assessment of population stratification between DP and PBD subgroups

To assess potential population stratification, we compared genotype distributions of the five candidate SNPs between DP and PBD controls. Four SNPs showed no significant differences (rs613491: *P* = 0.689; rs620977: *P* = 0.222; rs3819288: *P* = 0.336; rs1131170: *P* = 0.245; **Supporting Information**: **Supplementary Table 4**), supporting genetic homogeneity between subgroups. However, rs605219 exhibited significant heterogeneity (*P* < 0.001), with higher CC genotype frequency in DP (27.7%) compared to PBD (9.0%). This discrepancy likely reflects selection bias due to differential recruitment sources (tertiary hospital dialysis centers vs. rural village blood donors) rather than true population stratification, as rs605219 is not a known ancestry-informative marker. To address this, we: (1) adjusted for route of infection in all multivariable models; (2) performed stratified analyses by population subgroup (**Table 3**); and (3) confirmed that rs605219 was not associated with HCV susceptibility in either subgroup (DP: OR = 0.70, P = 0.044; PBD: OR = 0.93, P = 0.503), suggesting the observed heterogeneity does not confound our primary findings.

## 4 Discussion

In this study, we investigated the role of genetic variations in the *KIR3DL1/3DS1* and *HLA-B* genes in HCV infection by examining five candidate SNPs: *KIR3DL1/3DS1* rs613491, rs605219, rs620977, and *HLA-B* rs3819288, rs1131170. The data indicated that the CC genotype of *KIR3DL1/3DS1* rs613491 and the CC genotype of *HLA-B* rs1131170 were associated with increased susceptibility to HCV, while the AC genotype of *HLA-B* rs1131170 was associated with decreased susceptibility. Stratified analyses further confirmed the association between carrying these genotypes and HCV infection susceptibility in certain subgroups. Additionally, bioinformatics analysis suggested that variations at the rs1131170 locus may play a regulatory role in transcription and translation mechanisms. These observational associations do not establish causality, and the underlying biological mechanisms require further experimental investigation. Two salient features of this study are its large sample size and the adjustment for all potential confounding factors, including *IL28B* rs12979860 and *IL28B* rs8099917. Both single nucleotide polymorphisms have been shown to be significantly associated with HCV susceptibility and spontaneous viral clearance (Medina et al., 2023).

The reasons for selecting *KIR3DL1/3DS1* and *HLA-B* are their highly polymorphic interactions that can modulate the effector functions of natural killer (NK) cells and some T cells. This genetically determined diversity affects the severity of infections, immune-mediated diseases, and some cancers, and impacts the course of immunotherapies, including transplantation. KIR3DL1 is an inhibitory receptor, and KIR3DS1 is an activating receptor encoded by the *KIR3DL1/3DS1* gene, which has more than 200 diverse and divergent alleles (Harrison et al., 2022). KIR3DL1 binds specifically to subgroups of HLA-A or HLA-B, which have a five-amino-acid motif on their outside α1-helix called Bw4 (Vivian et al., 2011). The expression of KIR3DL1 endows NK cells with the ability to detect diseased cells that may have lost or altered the expression of these HLA class I molecules (Malmberg et al., 2017; Boudreau and Hsu, 2018). KIR3DL1 may also act as an immune checkpoint inhibitor for functionally mature T cells (Parsons et al., 2010). The polymorphism of *KIR3DL1*, as well as the polymorphism within and outside the *Bw4* motif of *HLA*, affects the specificity and strength of the interaction. Polymorphism also determines the receptor’s expression level or signal transduction capacity (Kim et al., 2008; Saunders et al., 2016; Saunders et al., 2021). *KIR3DS1* and *KIR3DL1* are considered alleles of the same locus. Compared with its inhibitory counterpart *KIR3DL1*, *KIR3DS1* represents the only activating receptor with three extracellular domains, characterized by a short cytoplasmic tail and a positively charged residue within the transmembrane domain that allows recruitment of the ITAM-carrying adaptor molecule DAP12 (Körner and Altfeld, 2012). The *HLA-B* gene is part of the human leukocyte antigen (HLA) complex, located on chromosome 6. The HLA complex is divided into three subregions: class I, class II, and class III. The *HLA-B* gene belongs to the class I genes, encoding class I molecules on the cell surface together with *HLA-A* and *HLA-C*. These molecules are responsible for presenting endogenous peptides to CD8+ T cells, thereby assisting the immune system in identifying pathogens (Barbarino et al., 2015). The polymorphism of the *HLA-B* gene is not only closely related to the susceptibility and disease progression of HIV infection but also associated with Graves’ disease, nasopharyngeal cancer, rheumatoid arthritis, and other conditions (Aksak-Wąs et al., 2021). The HLA-B*13:02 and HLA-B*40:01 alleles have been implicated in susceptibility to SARS-CoV-2 infection, potentially attributable to their distinctive peptide anchoring motifs (Wang et al., 2023). Therefore, this study selected *KIR3DL1*/*3DS1* and *HLA-B* as the research subjects and employed a specific screening strategy to select the candidate SNPs.

In the logistic regression analysis, we found that individuals carrying the rs1131170 CC genotype of *HLA-B* were more susceptible to HCV infection, while those carrying the *HLA-B* rs1131170 AC genotype exhibited reduced susceptibility to HCV infection. The protective effect observed in AC genotype carriers may reflect the phenomenon of “heterozygote advantage” at this locus. Previous studies have shown that heterozygosity at *HLA-DRB1* rs660895 is associated with a decreased risk of rheumatoid arthritis (Das et al., 2018). HLA heterozygosity broadens the peptide repertoire presented by class I molecules, thereby enhancing T-cell clonal breadth and improving virological control; this quantitative increase in peptide diversity constitutes the molecular basis of heterozygote advantage by imposing stronger pathogen-mediated selective pressure and lowering viral load (Arora et al., 2020). Taken together, we speculate that the protective effect of the rs1131170 AC genotype stems from a quantitative or qualitative improvement in antigen presentation, thereby reducing susceptibility to HCV infection.

The rs613491 SNP of *KIR3DL1*/*3DS1* is located in the intronic region of the gene and does not directly encode proteins. However, intronic SNPs may alter transcription factor binding sites, thereby influencing gene transcriptional activity. For example, E.V. Antontseva et al. reported that rs174575, located in the first intron of the *FADS2* gene, is associated with overexpression of *FADS2*, as the G allele modulates gene expression by affecting the binding of the transcription factor E2F1 (Antontseva et al., 2023). Intronic polymorphisms can influence gene expression and phenotype by affecting transcriptional function. Dong et al. reported that an intronic SNP associated with central obesity acts as a transcription factor (TF) that binds and enhances molecular activity, increasing BCL2 expression (Dong et al., 2021). Grossi et al. demonstrated that an intronic SNP binds to FOXO3, enabling FOXO3 expression in response to diverse stress stimuli (Grossi et al., 2018). Another significant SNP, rs1131170, is located in an exon of *HLA-B* and represents a missense mutation. SNPinfo prediction results suggest that rs1131170 may function as an exonic splicing enhancer/silencer (ESE/ESS) or a nonsynonymous SNP (nsSNP), participating in the regulation of *HLA-B* gene expression. Thus, mutations in rs1131170 may alter splicing factor binding sites, affecting mRNA splicing processes. Such changes could lead to exon inclusion or skipping, thereby influencing protein structure and function. Alternatively, they may modify the secondary structure of *HLA-B* mRNA, such as forming or disrupting G-quadruplexes or hairpin structures, which can regulate translation efficiency and mRNA stability (Georgakopoulos-Soares et al., 2022). Additionally, the RNAfold web server was used to predict the secondary structure of rs1131170 using the minimum free energy (MFE) algorithm. The MFE algorithm posits that the most stable RNA secondary structure occurs when free energy is minimized. Differences in nucleotide sequences can alter RNA secondary structure due to changes in base-pairing energy. For rs1131170, the C allele exhibits lower MFE than the A allele, indicating differences in mRNA secondary structure stability.

Moreover, in seven cell lines, rs613491 is located at a peak of H3K4Me1, suggesting that this region may function as a super-enhancer. Super-enhancers typically consist of multiple enhancer elements occupied by key transcription factors and coactivators and are highly acetylated at histone H3K27. Variants in rs613491 may affect the activity of these enhancers, thereby modulating *KIR3DL1*/*3DS1* gene expression. rs613491 is also located at a peak of H3K27Ac, which is associated with active gene transcription. Variants in rs613491 may influence *KIR3DL1/3DS1* gene expression by altering H3K27Ac levels. Such changes in expression levels could affect natural killer (NK) cell function, thereby impacting susceptibility to HCV infection. Regions with H3K4Me1 and H3K27Ac peaks are often linked to chromatin accessibility. Variants in rs613491 may affect chromatin openness, influencing the binding of transcription factors and coactivators and subsequently altering *KIR3DL1/3DS1* gene expression (Kang et al., 2021). Given that aberrant histone modifications can drive abnormal gene expression patterns, they may play a role in the pathogenesis of inflammatory diseases and various cancers (Beacon et al., 2021). Furthermore, H3K4Me3 and H3K27Ac primarily accumulate at sites of active gene transcription, promoting and amplifying this critical biological process. Elevated H3K4Me3 levels have been linked to clinical prognosis in malignancies such as liver and cervical cancer (Li et al., 2018; Xiao et al., 2023). Considering that KIR3DL1 is an inhibitory receptor and KIR3DS1 is an activating receptor on NK cell surfaces, these SNPs may potentially influence the inhibitory or activating capacity of NK cells by upregulating *KIR3DL1/3DS1* expression, thereby potentially modulating innate immune mechanisms and influencing susceptibility to HCV infection.

The absence of significant associations between *KIR3DL1/3DS1、HLA-B* variants and spontaneous clearance carries important implications. First, this null finding suggests that the genetic determinants of initial infection susceptibility may differ mechanistically from those governing viral clearance. While KIR-HLA interactions regulate NK cell recognition of infected hepatocytes during acute infection, sustained viral control likely depends more heavily on adaptive T cell responses and IL28B-mediated interferon pathways. Second, the modest sample size of the spontaneous clearance group (n=311) limits statistical power; assuming an OR of 2.0 and minor allele frequency of 0.30, the power to detect significant associations at α=0.05 was approximately 65%, increasing the risk of Type II error. Third, the borderline protective effect of rs1131170 CC in the clearance analysis (OR = 0.62, P = 0.034, uncorrected) may reflect genuine biological heterogeneity, whereby the same genotype that increases infection risk paradoxically facilitates subsequent viral control—consistent with the “heterozygote advantage” hypothesis requiring larger samples to resolve. Moreover, the high proportion of missing HCV genotype data and the predominance of mixed infections in our cohort limit definitive conclusions regarding viral genetic effects on clearance.

Based on age, sex and high-risk populations revealed that the rs613491-C and rs1131170-C alleles increased susceptibility to HCV infection in certain subgroups. With advancing age, the gradual decline in immune system function weakens the ability to clear HCV. SNP variations may further impair this capacity, increasing susceptibility to HCV infection (Saxena and Panhotra, 2004). In females, estradiol and estrogen receptors appear to provide a degree of protection by modulating both innate and adaptive immune responses (Fish, 2008). Among individuals with mild liver function abnormalities, SNPs may influence NK cell-mediated immune surveillance of infected hepatocytes by regulating the affinity between *KIR3DL1/3DS1* and *HLA-Bw4 (*Khakoo et al., 2004). Renal dialysis patients typically exhibit compromised immune function, rendering them more vulnerable to HCV infection. SNP variations may further diminish NK cell activity, elevating the risk of HCV infection (Saxena and Panhotra, 2004). Additionally, considering that different routes of infection may lead to varying HCV inoculum sizes and immune response intensities, the rs1131170-C allele was associated with increased susceptibility in repeat blood donors. Collectively, these findings suggest complex interactions among age, sex, high-risk populations, and genetic factors.

Several limitations of this study should be acknowledged. First, all participants had a history of paid blood donation or medical procedures prior to the implementation of China’s Blood Donation Law in 1998, which may introduce selection bias related to healthcare access and socioeconomic status. Second, precise information regarding the timing of initial HCV infection and the cumulative volume of blood transfusion was difficult to obtain retrospectively, precluding assessment of dose-response relationships. Third, while we attempted to minimize selection bias by deriving all study groups from the same source population, potential population stratification remains a concern. To address this, we compared genotype distributions of the five candidate SNPs between dialysis patients (DP) and paid blood donors (PBD). Four SNPs showed comparable distributions (rs613491: *P* = 0.689; rs620977: *P* = 0.222; rs3819288: *P* = 0.336; rs1131170: *P* = 0.245; **Supporting Information**: **Supplementary Table 4**), supporting genetic homogeneity between subgroups. However, rs605219 exhibited significant heterogeneity (*P* < 0.001), with higher CC genotype frequency in DP (27.7%) compared to PBD (9.0%). This discrepancy likely reflects selection bias from differential recruitment sources (tertiary hospital dialysis centers vs. rural village blood donors) rather than true ancestral differences, as rs605219 is not a known ancestry-informative marker. We mitigated this potential confounding by: (a) adjusting for route of infection in all multivariable models; (b) performing subgroup-stratified analyses (**Table 3**); and (c) confirming consistent effect directions for our primary findings (rs613491 and rs1131170) across both subgroups. Regarding population generalizability, our findings are based on two highly specific high-risk populations representing extreme exposure scenarios. The predominance of HCV genotype 1 and mixed infections in our cohort (**Table 1**) may further limit applicability to regions with different viral genotype distributions. Caution should be exercised when extrapolating these associations to the broader Chinese population or to low-risk settings without independent validation.

Additionally, we acknowledge two important methodological limitations. First, given the known biological interaction between *KIR3DL1* and *HLA-Bw4* alleles, direct analysis of Bw4 status would have strengthened our study; however, our genotyping approach did not enable definitive identification of all Bw4-associated alleles. Moreover, due to the small frequencies of specific combined genotypes (n<5), we were unable to formally test gene-gene interactions (epistasis) between KIR and HLA variants. Future studies incorporating high-resolution HLA typing and larger cohorts are warranted to explore KIR-HLA-Bw4 interactions. Second, the elevated OR values observed for rs1131170 CC (OR = 4.20) may partly reflect case-control sampling in high-risk populations and subgroup size imbalance. We adjusted for various confounding factors—including age, sex and infection routes—using logistic regression. Notably, the genotypes of *IL28B* rs12979860 and rs8099917 were also incorporated into the adjustment model (**Supporting Information**: **Supplementary Table 5**). These effect estimates should be interpreted as measures of association strength rather than precise population parameters, and validation in larger, population-based cohorts is needed to confirm the magnitude of these effects.

## 5 Conclusion

In conclusion, our findings demonstrate an association between *KIR3DL1/3DS1/HLA-B* genetic variants and susceptibility to HCV infection in these high-risk Chinese populations. These genetic markers may modulate innate immune responses. Further functional studies are warranted to validate the underlying biological mechanisms.

## Data Availability

All aggregated genotype frequencies, summary statistics, and other data necessary to replicate the findings and verify the conclusions presented in this paper are available from the corresponding author upon reasonable request. The raw research data have been securely archived in a certified domestic repository in China in accordance with relevant regulations, and access is restricted exclusively to authorized Chinese researchers who have obtained the required regulatory approvals.
