# Supplementary material for: Impact of KIR3DL1/3DS1 and HLA-B polymorphisms on hepatitis C virus infection: a case-control study
Source: Front Cell Infect Microbiol. 2026 May 12;16:1801310. doi: 10.3389/fcimb.2026.1801310 (PMC13201228; doi:10.3389/fcimb.2026.1801310)
Supplement: Supplementary file 1 [file DataSheet1.docx]

***Supplementary Material***

**Table 1.** The basic information for the candidate SNPs

| Gene | SNPs | Allele | Regulome DBscores | SNPinfo function | | | |
| --- | --- | --- | --- | --- | --- | --- | --- |
|  |  |  |  | TFBS | Splicing | miRNA | nsSNP |
| *KIR3DL1/S1* | rs605219 | T＞C | 1f | / | Yes | / | Yes |
| *KIR3DL1/S1* | rs613491 | T＞C | 1f | / | / | / | / |
| *KIR3DL1/S1* | rs620977 | A＞T | 1f | / | / | / | / |
| *HLA-B* | rs3819288 | T＞C | 1f | / | / | / | / |
| *HLA-B* | rs1131170 | A＞C | 1f | / | Yes | / | Yes |

**Table 2.** Probes and primers of investigated KIR, HLA-B and IFNL4 SNPs for TaqMan assay.

| Gene | SNPs (Allele) | MAFa/b | *P*c | TaqMan-MGB probe/primers sequences (5’-3’) |
| --- | --- | --- | --- | --- |
| *KIR3DL1/S1* | rs605219 | 0.407/0.600 | 0.077 | Probe-T:FAM-CACCATGTTGCTCAT-MGB |
|  | T＞C |  |  | Probe-C:VIC-AGCACCATGTCGC-MGB |
|  |  |  |  | Forward primer:AGGGCGCCAAATAACATCCT |
|  |  |  |  | Reverse primer:CACTCCCTCCCTCGATTCC |
| *KIR3DL1/S1* | rs613491 | 0.143/0.479 | 0.111 | Probe-T:FAM-CTTGCCTTCCCCG-MGB |
|  | T＞C |  |  | Probe-C:VIC-CTTGCCCTCCCCG-MGB |
|  |  |  |  | Forward primer:GCTCGGCCCACATTTCTG |
|  |  |  |  | Reverse primer:CTTGACTCAGGAAAGGGAATGAA |
| *KIR3DL1/S1* | rs620977 | 0.138/0.488 | 0.148 | Probe-A:FAM-CCTTTCATTCCCTTTC-MGB |
|  | A＞T |  |  | Probe-T:VIC-CCGGCCTTTCTTT-MGB |
|  |  |  |  | Forward primer:TGCTCGGCCCACATTTCT |
|  |  |  |  | Reverse primer:CCAGTCTTCACAGAGCTTGACTCA |
| *HLA-B* | rs3819288 | 0.265/0.280 | 0.477 | Probe-T:FAM-TCAAGGTGATACATCC-MGB |
|  | T＞C |  |  | Probe-C:VIC-CAAGGCGATACATC-MGB |
|  |  |  |  | Forward primer:CAGGACCCCAACACCACAAC |
|  |  |  |  | Reverse primer:CAGCACATGTGACAATGAAGGA |
| *HLA-B* | rs1131170 | 0.172/0.731 | 0.054 | Probe-T:FAM-TTTCTACACCTCCGTGTC-MGB |
|  | A＞C |  |  | Probe-G:VIC-TTTCTACACCGCCGTGT-MGB |
|  |  |  |  | Forward primer:TCTCAGCCCCTCCTCACC |
|  |  |  |  | Reverse primer:CACGTAGCCCACTGAGATGAAG |
| *IFNL4* | rs12979860 | 0.050/0.080 | 0.090 | Probe-T: FAM-TCCCCGAAGGGTGA-MGB |
|  | C>T |  |  | Probe-C: VIC-CGAAGGCGCGAAC-MGB |
|  |  |  |  | Forward primer: TGCCTGTCGTGTACTGAACCA |
|  |  |  |  | Reverse primer: GAGCGCGGAGTGCAATTC |
| *IFNL4* | rs8099917 | 0.060/0.076 | 0.05 | Probe-T: FAM-TGAGCAATTTCACC-MGB |
|  | T>G |  |  | Probe-G: VIC-TGAGCAATGTCACCC-MGB |
|  |  |  |  | Forward primer: CAATTTGTCACTGTTCCTCCTTTTG |
|  |  |  |  | Reverse primer: TAAAGATGTGGGAGAATGCAAATGA |

Abbreviations: KIR, killer cell immunoglobulin-like receptor; HLA-B, human leukocyte antigen class I, B; IFNL4, interferon lambda 4; SNPs, single nucleotide polymorphisms; MAF, minor allele frequency.

^a^ minor allele frequencies in control group (Group A).

^b^ minor allele frequencies from HapMap of East Asian (EAS). (available at <https://www.ncbi.nlm.nih.gov/snp> ).

^c^*P* value of Hardy-Weinberg equilibrium for SNPs among control group (Group A).

**Table 3.** Hardy-Weinberg equilibrium test for rs613491 and rs1131170 among control group in the DP and PBD subgroups.

| Gene | SNPs | Subgroups | *P* |
| --- | --- | --- | --- |
| *KIR3DL1/S1* | rs613491 | DP | 0.718 |
|  |  | PBD | 0.090 |
| *HLA-B* | rs1131170 | DP | 0.155 |
|  |  | PBD | 0.201 |

Abbreviations: KIR, killer cell immunoglobulin-like receptor; HLA-B, human leukocyte antigen class I, B; SNPs, single nucleotide polymorphisms; PBD: paid blood donors; DP: dialysis patients.

*P* value of Hardy-Weinberg equilibrium for SNPs among control group (Group A).

**Table 4.** Assessment of population stratification: Genotype distributions of *KIR3DL1/3DS1* and *HLA-B* SNPs between dialysis patients and paid blood donors in the control group

| SNPs | genotype | DP (n=325) | PBD (n=700) | *P* |
| --- | --- | --- | --- | --- |
| *KIR3DL1/S1* rs613491 | TT | 233(71.7%) | 522(74.6%) | 0.689 |
|  | TC | 80(24.6%) | 157(22.4%) |  |
|  | CC | 8(2.5%) | 19(2.7%) |  |
| *KIR3DL1/S1* rs605219 | TT | 144(44.3%) | 197(28.1%) | ＜0.001 |
|  | TC | 86(26.5%) | 427(61.0%) |  |
|  | CC | 90(27.7%) | 63(9.0%) |  |
| *KIR3DL1/S1* rs620977 | AA | 235(72.3%) | 516(73.7%) | 0.222 |
|  | AT | 88(27.1%) | 166(23.7%) |  |
|  | TT | 2(0.6%) | 12(1.7%) |  |
| *HLA-B* rs3819288 | TT | 181(55.7%) | 364(52.0%) | 0.336 |
|  | TC | 112(34.5%) | 270(38.6%) |  |
|  | CC | 27(8.3%) | 48(6.9%) |  |
| *HLA-B* rs1131170 | AA | 216(66.5%) | 496(70.9%) | 0.245 |
|  | AC | 93(28.6%) | 181(25.9%) |  |
|  | CC | 16(4.9%) | 23(3.3%) |  |

Note: *P* values were calculated using Pearson chi-square test for genotype frequency distributions between subgroups. Bolded text represents substantially significant outcomes.

Abbreviations: SNP, single nucleotide polymorphism; DP, dialysis patients; PBD, paid blood donors; KIR, killer-cell immunoglobulin-like receptors; HLA, human leukocyte antigen.

**Table 5-1.** Adjusted regression coefficients for *HLA-B* rs1131170 across codominant, recessive and additive genetic models

| Gene | Variables | B | SE | Wald | *P* | Exp(B) | 95% CI lower limit | 95% CI upper limit |
| --- | --- | --- | --- | --- | --- | --- | --- | --- |
| *HLA-B* rs1131170 | Codominant model |  |  |  |  |  |  |  |
|  | population | 0.694 | 0.072 | 91.761 | ＜0.001 | 2.003 | 1.737 | 1.737 |
|  | sex | 0.265 | 0.113 | 5.534 | 0.019 | 1.304 | 1.045 | 1.045 |
|  | age | -0.034 | 0.005 | 41.261 | ＜0.001 | 0.966 | 0.956 | 0.956 |
|  | rs12979860 | 0.616 | 0.274 | 5.064 | 0.024 | 1.852 | 1.083 | 1.083 |
|  | rs8099917 | -0.627 | 0.263 | 5.685 | 0.017 | 0.534 | 0.319 | 0.319 |
|  | AA | - | - | 67.665 | ＜0.001 | \|  \| 1.000(ref) \| \| --- \| --- \| | - | - |
|  | AC | -0.456 | 0.127 | 12.802 | ＜0.001 | 0.634 | 0.494 | 0.814 |
|  | CC | 1.435 | 0.209 | 46.952 | ＜0.001 | 4.201 | 2.786 | 6.333 |
|  | Recessive model |  |  |  |  |  |  |  |
|  | population | 0.711 | 0.072 | 96.716 | ＜0.001 | 2.035 | 1.767 | 2.035 |
|  | sex | 0.262 | 0.112 | 5.430 | 0.020 | 1.299 | 1.042 | 1.299 |
|  | age | -0.035 | 0.005 | 43.739 | ＜0.001 | 0.966 | 0.956 | 0.966 |
|  | rs12979860 | 0.568 | 0.273 | 4.343 | 0.037 | 1.766 | 1.034 | 1.766 |
|  | rs8099917 | -0.609 | 0.262 | 5.391 | 0.020 | 0.544 | 0.326 | 0.544 |
|  | Recessive (CC vs AA+AC) | 1.544 | 0.208 | 55.154 | ＜0.001 | 4.682 | 3.115 | 4.682 |
|  | Additive model |  |  |  |  |  |  |  |
|  | population | 0.689 | 0.071 | 94.507 | ＜0.001 | 1.991 | 1.733 | 2.288 |
|  | sex | 0.249 | 0.111 | 5.065 | 0.024 | 1.283 | 1.033 | 1.593 |
|  | age | -0.034 | 0.005 | 43.159 | ＜0.001 | 0.966 | 0.956 | 0.976 |
|  | rs12979860 | 0.499 | 0.268 | 3.456 | 0.063 | 1.647 | 0.973 | 2.787 |
|  | rs8099917 | -0.584 | 0.257 | 5.167 | 0.023 | 0.558 | 0.337 | 0.923 |
|  | Additive (per C allele) | 0.291 | 0.080 | 13.222 | ＜0.001 | 1.338 | 1.144 | 1.566 |

Note: B, unstandardized regression coefficient; SE, standard error; Wald, Wald chi-square statistic; P, P-value; Exp(B), odds ratio (OR); 95% CI, 95% confidence interval. All models were adjusted for population (route of infection), sex, age, *IL28B* rs12979860, and *IL28B* rs8099917. Reference categories: AA genotype for codominant model; AA+AC genotypes for recessive model; per-allele effect for additive model. Bold type indicates statistically significant associations (*P* < 0.05).

Abbreviations: HLA, human leukocyte antigen; HCV, hepatitis C virus; IL28B, Interleukin 28B; CI, confidence interval.

**Table 5-2.** Adjusted regression coefficients for *KIR3DL1/S1* rs613491 across codominant, recessive and additive genetic models

| Gene | Variables | B | SE | Wald | *P* | Exp(B) | 95% CI lower limit | 95% CI upper limit |
| --- | --- | --- | --- | --- | --- | --- | --- | --- |
| *KIR3DL1/S1* rs613491 | Codominant model |  |  |  |  |  |  |  |
|  | population | 0.656 | 0.070 | 88.977 | ＜0.001 | 1.927 | 1.681 | 2.208 |
|  | sex | 0.244 | 0.110 | 4.938 | 0.026 | 1.276 | 1.029 | 1.582 |
|  | age | -0.033 | 0.005 | 39.988 | ＜0.001 | 0.968 | 0.958 | 0.978 |
|  | rs12979860 | 0.527 | 0.266 | 3.943 | 0.047 | 1.694 | 1.007 | 2.851 |
|  | rs8099917 | -0.494 | 0.253 | 3.821 | 0.051 | 0.610 | 0.372 | 1.001 |
|  | TT | - | - | 19.966 | ＜0.001 | \|  \| 1.000(ref) \| \| --- \| --- \| | - | - |
|  | TC | 0.055 | 0.117 | 0.218 | 0.640 | 1.056 | 0.839 | 1.329 |
|  | CC | 1.142 | 0.256 | 19.964 | ＜0.001 | 3.132 | 1.898 | 5.168 |
|  | Recessive model |  |  |  |  |  |  |  |
|  | population | 0.655 | 0.070 | 88.919 | ＜0.001 | 1.926 | 1.681 | 2.207 |
|  | sex | 0.245 | 0.110 | 4.977 | 0.026 | 1.277 | 1.030 | 1.583 |
|  | age | -0.033 | 0.005 | 39.954 | ＜0.001 | 0.968 | 0.958 | 0.978 |
|  | rs12979860 | 0.527 | 0.265 | 3.947 | 0.047 | 1.694 | 1.007 | 2.851 |
|  | rs8099917 | -0.495 | 0.252 | 3.850 | 0.050 | 0.609 | 0.372 | 0.999 |
|  | Recessive (CC vs AA+AC) | 1.128 | 0.254 | 19.751 | ＜0.001 | 3.090 | 1.879 | 5.081 |
|  | Additive model |  |  |  |  |  |  |  |
|  | population | 0.649 | 0.069 | 87.791 | ＜0.001 | 1.913 | 1.670 | 2.191 |
|  | sex | 0.242 | 0.109 | 4.880 | 0.027 | 1.273 | 1.028 | 1.577 |
|  | age | -0.033 | 0.005 | 39.914 | ＜0.001 | 0.968 | 0.958 | 0.978 |
|  | rs12979860 | 0.497 | 0.264 | 3.528 | 0.060 | 1.643 | 0.979 | 2.760 |
|  | rs8099917 | -0.466 | 0.251 | 3.445 | 0.063 | 0.628 | 0.384 | 1.026 |
|  | Additive (per C allele) | 0.297 | 0.089 | 11.175 | ＜0.001 | 1.346 | 1.131 | 1.602 |

Note: B, unstandardized regression coefficient; SE, standard error; Wald, Wald chi-square statistic; P, P-value; Exp(B), odds ratio (OR); 95% CI, 95% confidence interval. All models were adjusted for population (route of infection), sex, age, IL28B rs12979860, and IL28B rs8099917. Reference categories: AA genotype for codominant model; AA+AC genotypes for recessive model; per-allele effect for additive model. Bold type indicates statistically significant associations (*P* < 0.05).

Abbreviations: KIR, killer-cell immunoglobulin-like receptors; HCV, hepatitis C virus; IL28B, Interleukin 28B; CI, confidence interval.

**Table 6.** Association between candidate SNPs and HCV spontaneous clearance, with and without HCV genotype adjustment

| Gene | SNPs(genotype) | Group B n (%) n=311 | Group C n (%) n=545 | OR (95%CI)^a^ | *P*^a^ | OR (95%CI)^b^ | *P*^b^ |
| --- | --- | --- | --- | --- | --- | --- | --- |
| *KIR3DL1/3DS1* | rs613491 |  |  |  |  |  |  |
|  | TT | 213(71.7) | 357(69.2) | 1 |  | 1 |  |
|  | TC | 66(22.2) | 122(23.6) | 1.10(0.77-1.57) | 0.589 | 1.31(0.49-3.48) | 0.591 |
|  | CC | 18(6.1) | 37(7.2) | 1.59(0.86-2.95) | 0.143 | 2.27(0.67-7.75) | 0.190 |
|  | Dominant model |  |  | 1.20(0.86-1.66) | 0.282 | 1.61(0.70-3.69) | 0.260 |
|  | Recessive model |  |  | 1.55(0.84-2.86) | 0.161 | 2.14(0.64-7.17) | 0.216 |
|  | Additive model |  |  | 1.19(0.93-1.53) | 0.163 | 1.46(0.84-2.54) | 0.185 |
| *KIR3DL1/3DS1* | rs605219 |  |  |  |  |  |  |
|  | TT | 87(30.7) | 167(34.6) | 1 |  | 1 |  |
|  | TC | 168(59.4) | 276(57.1) | 0.78(0.56-1.10) | 0.158 | 0.65(0.27-1.58) | 0.345 |
|  | CC | 28(9.9) | 40(8.3) | 0.71(0.40-1.28) | 0.252 | 0.32(0.10-0.99) | 0.047 |
|  | Dominant model |  |  | 0.77(0.56-1.08) | 0.126 | 0.52(0.23-1.16) | 0.111 |
|  | Recessive model |  |  | 0.82(0.48-1.42) | 0.488 | 0.39(0.14-1.10) | 0.074 |
|  | Additive model |  |  | 0.82(0.63-1.06) | 0.128 | 0.58(0.34-1.00) | 0.049 |
| *KIR3DL1/3DS1* | rs620977 |  |  |  |  |  |  |
|  | AA | 222(72.1) | 371(69.9) | 1 |  | 1 |  |
|  | AT | 84(27.3) | 159(29.9) | 1.21(0.87-1.67) | 0.254 | 1.28(0.58-2.82) | 0.549 |
|  | TT | 2(0.6) | 1(0.2) | 0.26(0.02-2.90) | 0.273 | - | - |
|  | Dominant model |  |  | 1.18(0.86-1.63) | 0.309 | 1.28(0.58-2.82) | 0.549 |
|  | Recessive model |  |  | 0.25(0.02-2.75) | 0.255 | - | - |
|  | Additive model |  |  | 1.14(0.84-1.57) | 0.402 | 1.28(0.58-2.82) | 0.549 |
| *HLA-B* | rs3819288 |  |  |  |  |  |  |
|  | TT | 156(51.5) | 290(55.6) | 1 |  | 1 |  |
|  | TC | 122(40.3) | 193(37.0) | 0.83(0.61-1.13) | 0.241 | 1.34(0.61-2.94) | 0.470 |
|  | CC | 25(8.3) | 39(7.5) | 0.82(0.47-1.44) | 0.494 | 0.66(1.57-2.80) | 0.574 |
|  | Dominant model |  |  | 0.83(0.62-1.11) | 0.213 | 1.20(0.57-2.52) | 0.640 |
|  | Recessive model |  |  | 0.89(0.52-1.53) | 0.671 | 0.59(0.14-2.38) | 0.454 |
|  | Additive model |  |  | 0.87(0.69-1.10) | 0.248 | 1.02(0.56-1.84) | 0.950 |
| *HLA-B* | rs1131170 |  |  |  |  |  |  |
|  | AA | 193(66.1) | 368(73.2) | 1 |  | 1 |  |
|  | AC | 47(16.1) | 81(16.1) | 0.92(0.61-1.40) | 0.704 | 1.36(0.52-3.61) | 0.533 |
|  | CC | 52(17.8) | 54(10.7) | 0.62(0.40-0.97) | 0.034 | 1.48(0.54-4.07) | 0.447 |
|  | Dominant model |  |  | 0.77(0.56-1.07) | 0.120 | 1.42(0.64-3.14) | 0.390 |
|  | Recessive model |  |  | 0.63(0.41-0.97) | 0.037 | 1.35(0.51-3.55) | 0.542 |
|  | Additive model |  |  | 0.81(0.66-1.00) | 0.047 | 1.23(0.76-2.02) | 0.401 |

Note: *P* Value, OR, and 95% CIs of ^(a)^were computed on the basis of the logistic regression model, adjusted by sex, age, *IL28B* rs12979860, *IL28B* rs8099917, and infection route. OR, and 95% CIs of ^(b)^were computed on the basis of the logistic regression model, adjusted by sex, age, *IL28B* rs12979860, *IL28B* rs8099917, HCV genotype and infection route. Bolded text represents substantially significant outcomes.

Abbreviations: Group B,spontaneous clearance group ; Group C, persistent infection group; HLA, human leukocyte antigen; IL28B, Interleukin 28B; KIR, killer‐cell immunoglobulin‐like receptors; SNPs, single nucleotide polymorphisms.
